# Structure–Activity Relationships and Changes in the Inhibition of Xanthine Oxidase by Polyphenols: A Review

**DOI:** 10.3390/foods13152365

**Published:** 2024-07-26

**Authors:** Kexin Li, Yumei Wang, Wanlu Liu, Chengfeng Zhang, Yu Xi, Yanv Zhou, He Li, Xinqi Liu

**Affiliations:** 1 Key Laboratory of Geriatric Nutrition and Health, Beijing Technology and Business University, Beijing 100048, China; 2230202116@st.btbu.edu.cn (K.L.); wym110809@126.com (Y.W.); liuwanlu19990115@163.com (W.L.);; 2 The Product Makers Co., Ltd., Shanghai 200444, China

**Keywords:** xanthine oxidoreductase, polyphenol, structure–activity relationship, processing, digestion, synergistic effect

## Abstract

Hyperuricemia (HUA), or elevated uric acid in the blood, has become more prevalent in recent years. Polyphenols, which are known to have good inhibitory activity on xanthine oxidoreductase (XOR), are effective in uric acid reduction. In this review, we address the structure–activity relationship of flavonoids that inhibit XOR activity from two perspectives: the key residues of XOR and the structural properties of flavonoids. Flavonoids’ inhibitory effect is enhanced by their hydroxyl, methoxy, and planar structures, whereas glycosylation dramatically reduces their activity. The flavonoid structure–activity relationship informed subsequent discussions of the changes that occur in polyphenols’ XOR inhibitory activity during their extraction, processing, gastrointestinal digestion, absorption, and interactions. Furthermore, gastrointestinal digestion and heat treatment during processing can boost the inhibition of XOR. Polyphenols with comparable structures may have a synergistic effect, and their synergy with allopurinol thus provides a promising future research direction.

## 1. Introduction

Xanthine oxidoreductase (XOR), a flavin protease common in mammals, irreversibly catalyzes the final reaction of uric acid metabolism, during which hypoxanthine and xanthine are converted into uric acid [1]. XOR is one of the key therapeutic targets for elevated uric acid, termed hyperuricemia (HUA), because of the key rate-limiting steps in purine metabolism. An abnormal metabolic syndrome, HUA is caused by a purine metabolism disorder and is manifested by flares in serum uric acid (SUA) levels that exceed 420 umol/L twice in the same day. In severe cases, uric acid crystals can also be deposited in the kidneys, causing acute kidney disease, chronic interstitial nephritis, or kidney stones [2,3]. HUA has, thus, received considerable scientific and medical scrutiny in recent years, providing ample evidence that the disorder is an independent risk factor for chronic kidney disease, hypertension, cardiovascular and cerebrovascular diseases, and diabetes, among others [4,5]. The prevalence of hyperuricemia has risen globally in the last decade. One study investigated the prevalence of gout in five countries and found an increasing trend. Prevalence rates ranged from <1% to 6.8% across countries, with overall prevalence rates particularly high in developed countries such as the United States and Australia [6,7]. The low percentage of patients receiving urate-lowering therapy after diagnosis (<50% in all cases) and poor adherence make it a metabolic disease that requires urgent attention [8].

Polyphenols are a class of substances containing one or more hydroxyl groups based on the benzene ring structure, and have a variety of biological activities [9,10,11]. Recent research has highlighted their XOR inhibitory activity and flexible structure [12,13]. Polyphenols can be roughly divided into flavonoids and non-flavonoids according to their number of benzene rings, substituents on benzene rings, and connections between benzene rings. Common amongst flavonoids is their 15-carbon skeleton with a C6-C3-C6 structure (composed of two aromatic A and B rings and oxygen-containing three-carbon C rings). They are subdivided into flavonoids, flavonols, flavanones, flavan-3-alcohols, and anthocyanins, according to the different substituents in different positions on different rings. The non-flavonoids are mainly derivatives of hydroxycinnamic acid and hydroxybenzoic acid, such as phenolic acid and stilbene [14,15,16]. Studies have found that flavonoids perform well in the inhibition of XOR activity, and that there is a certain link between this inhibitory activity and flavonoid structure; however, little has been ascertained about these conformational relationships. In addition, a variety of factors can affect the enzyme inhibitory activity of flavonoids from extraction to their in vivo action on the target, and these require consideration in practical applications.

For the development of natural products for hyperuricemia, it is important to find natural products rich in polyphenols with high XOR inhibitory activity. In this work, we discuss the structure–activity link between flavonoids and their ability to suppress XOR activity from two perspectives: the important XOR residues and the structural characteristics of the flavonoids present in polyphenols. In addition, various explanations of the alterations that occur in the XOR inhibitory activity of polyphenols during their processing, gastrointestinal digestion, and absorption, as well as their inter-polyphenol interactions, were explored based on structure–activity connections.

## 2. Structure–Activity Relationship of Flavonoid Inhibition of XOR: Two Perspectives

In their inhibition of XOR, flavonoids generally cause structural changes in the enzyme by binding to the active center of XOR, thereafter reducing the production of uric acid through competitive, non-competitive, or mixed inhibition. The various flavonoid monomers differ in their capacity to inhibit XOR, depending on their specific conformations. Therefore, a good understanding of structure–activity relationships is helpful for the efficient screening of enzyme inhibitors [17]. From the perspective of XOR and flavonoids, this section will focus on the important residues in enzymes and particular groups of flavonoids, as well as the structure–activity relationship of flavonoids in their inhibition of XOR.

### 2.1. From the Perspective of Enzymes: Contribution of Key Residues in Active Centers

**Figure 1:**
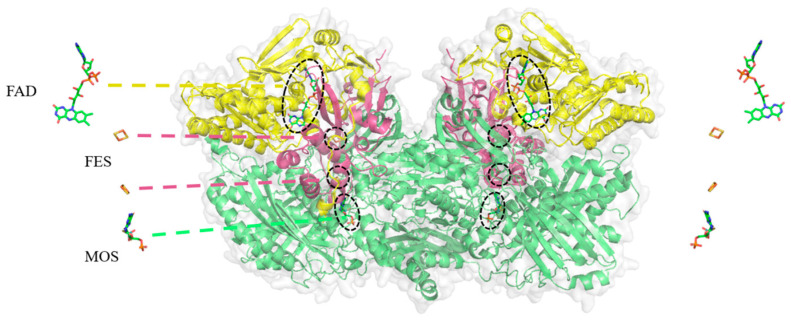
XOR structure: red iron-sulfur clusters represent the cofactor domain structure; yellow represents the flavin adenine dinucleotide (FAD) cofactor domain structure; green represents the molybdenum cofactor structure domain; protein structure from PDB: 3NVW. The black circles show the regions where the cofactors are located.

As shown in Figure 1, XOR is a homologous dimer made up of two identical polypeptide chains. It is a molybdenum-containing flavin protease with a molecular weight of roughly 300 kDa [18]. Each subunit in the enzyme has the following three cofactor domains: the N-terminal 20 kDa iron-sulfur cluster (2Fe/S) cofactor domain (residues 1–165), the approximately 40 kDa flavin adenine dinucleotide (FAD) cofactor domain (residues 226–531), and the 85 kDa molybdopterin cofactor (MOC) domain (residues 589–1332) [19]. XOR can accelerate the transformation of a variety of substrates and has a low level of substrate specificity. Purine, nitrate, and other substrates are often coupled to MOC in the center of molybdopterin. In the molybdenum center, the substrate xanthine is first oxidized and then hydroxylated to produce uric acid. Molybdenum (VI) becomes reduced to Mo (IV), whereafter electrons are reduced alongside the iron-sulfur cluster to the FAD center, resulting in ROS or NADH [18].

**Figure 2:**
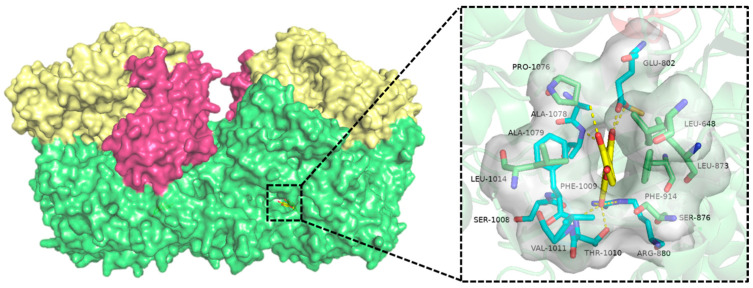
Schematic representation of X-ray crystal diffraction results of quercetin bound to XOR: The inset figure depicts how quercetin connects with the active pocket at a specific angle. The active pocket in the Moc’s active site is represented by the white translucent portion; green molecules represent the remaining amino acid residues in the active pocket; blue represents the amino acid residues that form hydrogen bonds with quercetin; yellow represents quercetin.

Hydrophobic residues at an active site determine the overall pattern of binding between substrate and enzyme. In Figure 2, for example, X-ray crystal diffraction of a quercetin/XOR complex shows that quercetin binds to XOR in a B-ring position, pointing towards the MOC active center of the enzyme. The van der Waals force formed between the conjugated ring structure of the quercetin and the phenylalanine residue at the active site of the enzyme, in conjunction with the hydrogen bond or polar interaction between the outer epoxide group or hydroxyl group of quercetin and polar residue, determine the mode in which quercetin binds to XOR. Ring A and ring C of quercetin are coplanar, the torsion angles of ring B and ring C are fixed by the π-π force between Phe914 and Phe1009, and the van der Waals force is formed between ring C and the hydrophobic residues Leu 873, Leu 1014, Leu 648, Val 1011, and Phe 1013 [20]. Additionally, the hydroxyl group on the ring interacts with the specific hydrogen bonds of the catalytic-related residues Arg 880 and Glu 802. The interaction patterns between other flavonoids and XOR were also analyzed and are presented in Table S1 [21,22,23,24,25,26,27,28,29,30,31].

Du et al. discovered that Glu residues are typically present close to the MOC active site of the molybdenum family, where they play an important role: when the substrate or inhibitor binds to the enzyme, they form a complex that facilitates orientation and accelerates binding, before also forming hydrogen bonds that support the binding between the substrate/inhibitor and enzyme [32]. Glu802 and Arg880 have been shown to play key roles in substrate binding to enzymes. Arg880 is the critical residue involved in the final protonation of uric acid and its release from the active site, and Glu802 facilitates substrate localization to the active center of the enzyme, also playing a key role in the stabilization of the negative charge that is generated in the transition state structure [33,34]. In their investigation of the conformational changes that occur in XOR when combined with allopurinol, soybean, and puerarin, Pan et al. discovered that during the binding process, amino acid residues Gly800–Glu802 showed obvious conformational changes following the addition of these three inhibitors, forming a potentially important residue [35]. Furthermore, Arg880 and Thr1010 are known to be located in the ‘pocket portion’ of the active site, and inhibitor binding may tighten this region, inhibiting substrate binding and improving inhibition. At the MOC site, Glu802, Arg880, Thr1010, Ser876, and Asn768 commonly establish hydrogen bonds with the hydroxyl groups of flavonoids during their binding with XOR, thus enhancing their affinity.

Key residues may also play various roles in the binding between polyphenols and XOR: (1) Prior to their binding, they can form complexes with substrates, which facilitates their orientation and promotes binding in some of the enzyme and polyphenol groups; (2) Key residues can also enhance the stability of polyphenol–enzyme complexes by creating hydrogen bonds with their hydrophobic substituents, such as hydroxyl groups, or by creating π-π interaction forces, usually Phe, with their benzopyrum rings; and (3) After the arrival of polyphenols at the active site of an enzyme, the conformational shift of certain residues tightens the active region, inhibiting the entry of the substrate.

### 2.2. From the Perspective of Polyphenols: Structure–Activity Relationship of Flavonoid Inhibition of XOR

#### 2.2.1. Hydroxylation

**Figure 3:**
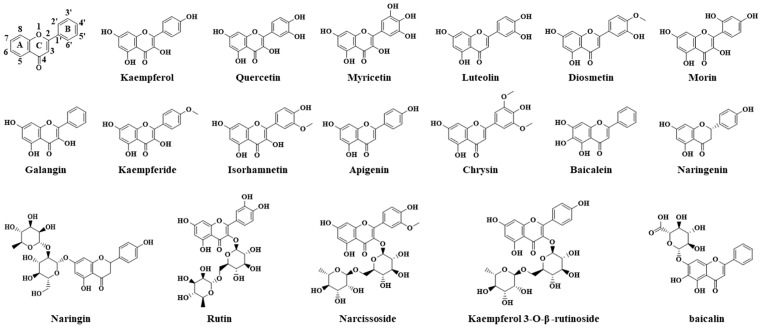
Diagrams of the structures of different flavonoids.

The hydroxylation that occurs on the core scaffold of flavonoids greatly affects their affinity with XOR and the extent of their inhibition of this enzyme (the structures of the polyphenols mentioned below are shown in Figure 3). The contribution of hydroxylation at the C5 and C7 positions of the A-ring to promote inhibitory activity has been confirmed in several studies [36]. In a comparative analysis of the structure and XOR inhibitory ability of various flavonoids, Cos et al. found that those with higher XOR inhibitory activity generally possess hydroxyl substituents at the 5 and 7 positions of ring A [37]. Similarly, Yuan et al. ascertained that almost all flavonoids in their study with good inhibitory activity have hydroxyl residues at their C5 and C7 sites and also found evidence of the role played by hydroxylation at C3 and C4 [38]. Furthermore, little distinction was found between the inhibitory activities of kaempferol (4′-OH), quercetin (3′,4′-OH), and myricetin (3′,4′,5′-OH), indicating that the extent of hydroxylation on the B ring has no significant effect. The difference in the inhibitory activities of the three polyphenols may be due to the instability of the polar hydroxyl group of the benzene ring B, which can extend to the hydrophobic region of the XOR active cavity and cause changes in binding capacity [39]. The contribution of hydroxyl to XOR inhibitory activity may, thus, be attributed to the fact that hydroxyl groups at special sites can form hydrogen bonds with key XOR residues, which improves the stability of the flavonoid’s binding to the enzyme’s Mo center and prevents the catalysis of substrates [40].

#### 2.2.2. Methoxylation

Methoxy group substitution has been shown to increase the polarity of flavonoids, thereby improving the forces of their interaction with hydrophobic residues inside the enzyme while also increasing binding ability. In one study, for instance, the methoxy group demonstrated greater activity than the hydroxyl group in a comparison between geranitin (C-4′-OCH_3_) and luteolin (C-4′-OH), while in the comparison of kaempferin (C-4′-OCH_3_) and alpinein (C-4′-H), it was demonstrated that the substitution of the methoxy group for the H group enhanced inhibitory activity [31]. Ou et al. speculated that methoxy substitution could increase hydrogen bonding sites, improve XOR binding affinity, and more effectively prevent substrate binding, which would explain the superiority of the inhibitory activity of kaempin compared to that of galangin [21]. Moreover, the conclusion of Yang et al. that the substituent of methoxy increases XOR inhibitory activity proved to also be valid for non-flavonoid chalcones, since they found that two methoxy derivatives of chalcone, (E)-1-(2,4-dimethoxyphenyl)-3-(4-propan-2-ylphenyl)prop-2-en-1-one, exhibited better XOR inhibitory activity than allopurinol [41].

However, the same methoxy alteration has also been reported to result in opposing modifications to inhibitory action. Different test methodologies provided conflicting outcomes in comparisons between galangin and kaempferin (C4′methoxy-substituted hydrogen group). The characteristic absorption peak of the substrate was found to be at 295 nm, which interferes with the measurement of uric acid production and could potentially cause deviations in data determined via spectrophotometry.

#### 2.2.3. Glycosylation

Naturally occurring flavonoids frequently combine with gyrogroups to create glycosides; however, numerous investigations have shown that the XOR inhibitory effect of flavonoids is significantly reduced or even eliminated following glycosylation. All of the glycosylated flavonoids in Zhao’s investigation, for example, were found to have only negligible inhibitory action (IC_50_ > 200 μM) and, according to Cavia-Saiz, the XOR inhibitory action of naringenin was drastically diminished following glycosylation [31]. Furthermore, among the flavonoids isolated from rhododendron extract for investigation by Deng, all with glycosylation at C-3 showed low inhibitory activity [42]. The addition of glycosyl units lengthens the molecular chains of flavonoids, increases their steric hindrance, and raises the possibility of mutual repulsion between the substances and the amino acid residues at an active site [43,44]. The destruction of the planar structure of flavonoids via glycosylation substitution makes it difficult for substances to access the active center of XOR and bind with amino acid residues, and their ability to inhibit is consequently reduced.

Interestingly, flavonoids with O-glucopyranoside in the C-7 site showed less of a decrease in xanthine oxidase inhibitory activity [45,46], possibly because glucopyranoside has less relative spatial site resistance to approach the active cavity and maintain the stability of glycoside–XOR interactions in the active site [47].

#### 2.2.4. Planar Structure

The π-π conjugation of C2=C3 with the C4 oxygen group plays an important role in maintaining the planar structure of flavonoids. Da Silva et al. compared the structure of several flavonoids (namely allopurinol, apigenin, quercetin, myricetin, isovitexin, genistein, and naringenin) with different XOR inhibitory activities using molecular mechanics (MM) and semi-empirical (AM1) methods. The results showed that the naringenin without a planar structure showed no inhibitory activity. Moreover, these researchers discovered that XOR inhibitory action decreased with increasing A ring-to-heteroxo ring torsion angle and, at a torsion angle greater than 27°, XOR inhibitory activity was lost entirely. A possible explanation for this phenomenon is that when the torsion angle exceeds 27°, the hydroxyl group on the phenol ring moves to the region of XOR where non-polar residues are present and is consequently unable to form a complex with the enzyme [48].

In summary, hydroxylation or methoxylation at special sites on the main ring can increase the enzyme inhibition ability of flavonoids, while glycosylation is not conducive to their binding with XOR. Furthermore, the hydrogenation of double bonds can destroy the structural stability of flavonoids and lead to a decrease in their inhibitory activity. Non-flavonoids, such as phenolic acids, chalcones, and other polyphenols, appear to reflect this regularity; however, few studies address this conjecture and it requires additional investigation.

## 3. Effects of Different Factors on the XOR Inhibitory Activity of Polyphenols

In the food industry, there are many stages of polyphenols, from their presence in plants to their final entry into the human body, where they are bound to xanthine oxidase. In this section, we have chosen to discuss three stages where polyphenols are highly variable (processing, gastrointestinal digestion and absorption, and finally synergistic binding to enzymes). Discussion of the changes in the inhibition of xanthine oxidase activity by polyphenols at these stages will hopefully provide theoretical support for the development of uric acid-lowering polyphenol products.

### 3.1. Effects of Processing

#### 3.1.1. Heat Treatment

The effect of processing on the ability of polyphenols to inhibit xanthine oxidase is shown in Table 1. To improve the bioactivity and availability of plant polyphenols, chemical hydrolysis, enzymatic hydrolysis, fermentation, heat treatment, and other processing methods are often employed, among which heat treatment provides the advantages of lower cost and fewer adverse effects. Moreover, heat treatment has been shown to enhance the XOR inhibitory activity of polyphenols. In Li’s study on the hydrothermal treatment of Flos Sophorae Immaturus, it was found that the inhibition rate of its flavonoid constituents could be increased from 32.42% to 89.00% by being treated at 220 °C for 30 min. The rapid conversion of the main components of rutin, narcissoside, and kaempferol-3-O-rutinoside to quercetin, kaempferol, isorhamnetin, and other aglycones at high temperatures has also been reported [49]. Similar conclusions have also been obtained from stir-frying, including the degradation, transformation, and interaction of flavonoids that occur during this type of heat treatment [50].

However, it should also be noted that excessively high processing temperatures can lead to the thermal degradation of polyphenols. Additionally, it has been shown that ultrasonic treatment also has a positive effect on sample preparation, and that ultrasonic assistance exhibits a synergistic effect with heat treatments and can enhance their effect, with ultrasonic power exerting a stronger influence than ultrasonic time [51,57].

Heat treatment can increase the inhibition of XOR by flavonoids, which is affected by two aspects of the food matrix and polyphenol molecules: (1) heat treatment increases the leaching rate of polyphenols from the cell wall, and a high temperature inactivates polyphenol oxidase, which increases the release of flavonoids from the food matrix from multiple perspectives [58]. (2) changes in flavonoids such as degradation, transformation, and interactions take place during heat treatment, glycosidic bonds are broken during the heat treatment, and the glycosylation is partially detached [59]. From the discussion of the conformational relationship of glycosylated flavonoids in the previous section, it is concluded that the remaining glycosidic portion has a small molecular structure and can enter and bind to the XOR active site, thus inhibiting substrate conversion and uric acid production.

Moreover, in the heat treatment of coffee, it was found that the thermal reaction products of caffeic acid conversion exerted the highest XOR inhibitory activity (>75%) at 170 °C; however, this inhibitory activity decreased significantly at higher temperatures (200 °C), suggesting that heat treatment can have a similar effect on non-flavonoid phenolic acids [52].

#### 3.1.2. Other Treatments

While extraction heating time and temperature have been shown to exert significant influences on the content of flavonoids, the choice of solvent and extraction method, as well as the solid-to-liquid ratio, is also known to affect XOR inhibitory activity in different types and contents of extracted polar polyphenols.

In terms of solution-to-solvent ratio, the maximum inhibition of XOR is observed when the solid/liquid ratios of ethanol to water are 1:30 and 1:20 (60.04% and 56.88%, respectively), and this inhibitory activity was directly correlated with the presence of different concentrations of caffeic acid, p-coumaric acid, chlorogenic acid, and isoquercetin [54]. However, in the extraction of Artemisia campestris dried leaf polyphenols, those extracted via solvent fractionation with ethyl acetate were shown to have the highest XOR inhibitory activity amongst various combinations of solvents and extraction methods, and this activity was positively correlated with the contents of total polyphenols and total flavonoids extracted (R^2^ = 0.56, R^2^ = 0.55). These findings may have been linked to the high contents of cirsiliol, cirsilineol, luteolin, kaempferol, and quinic acid in the extracts [55]. Shankaranarayana et al. found that the 80% ethanol extract of Baby Corn Silk exhibited the best xanthine oxidase inhibitory activity, which was 1.22, 1.85, and 3.02 times higher than the inhibitory activity of methanol, water, and acetone extracts, respectively. This inhibitory activity was significantly correlated with the total phenolic content [60].

High-pressure treatment is another common processing method, known to increase the extraction rate of polyphenols; however, it seems to have little effect on the improvement of XOR inhibition rates [56,61].

### 3.2. Effects of Digestion and Absorption on the XOR Inhibitory Activity of Polyphenols

Once consumed, polyphenols are first digested in the body by the oral cavity, stomach, and intestine, then absorbed by the intestinal epithelial cells, after which they enter the systemic circulation through the portal vein. During this process, interesting changes occur in the biological activity of polyphenols. The effect of digestion and absorption on the ability of polyphenols to inhibit xanthine oxidase is shown in Table 2.

#### 3.2.1. In Vitro Gastrointestinal Digestion

Polyphenols maintain high levels of stability during gastric digestion, and any minor increase in activity following gastric digestion is generally attributed to their release from the food substrate in the strongly acidic environment [62,63,64]. Major changes to polyphenols occur mainly during intestinal digestion. The slightly alkaline environment in the intestine exerts a great influence on polyphenols, during which flavonoids undergo deglycosylation, glucuronidation, methylation, sulfonation, and hydroxylation [65].

However, during in vitro digestion, the release and transformation of flavonoids generally had a facilitating effect on the changes in XOR inhibitory activity, and similar conclusions were observed with different raw materials. In a study of different stages of in vitro digestion of apple polyphenols, intestinal digestives exhibited higher levels of XOR inhibition than the extracts. This was probably due to the high recovery rates (150.94% and 90.97%) of total dihydrochalcone (primarily phenylpropyl) observed at this stage [56].

**Table 2:** Effects of digestion and absorption on xanthine oxidase inhibition by polyphenol extracts.

Polyphenol Source	Treatment	Total Polyphenols	Total Flavone	XOR Inhibition	Mechanism	Reference
Flos Sophorae Immaturus	Simulated gastrointestinal digestion	↑	↑	↑	The amount of bioabsorbable polyphenols, flavonoids, and phenolic acids increased	[66]
	Simulated intestinal absorption	↓	↓	↑	The content of phenolic compounds decreased slightly after dialysis, but phenolic compounds increased compared with the raw materials	
Wheat bread with onion skin added	Simulated gastrointestinal digestion	↑	-	↑	Gastrointestinal absorption may enrich OS-supplemented bread extracts in highly bioactive compounds	[67]
	Simulated intestinal absorption	↓	-	↓		
Purple sweet potato	Simulated gastric digestion	↓	-	-	The recovery of anthocyanin in stomach samples was 70%	[68]
	Simulated intestinal digestion	↓	-	↓	The change in pH in the intestinal environment from 2.0 to 7.5 leads to irreversible breakdown of anthocyanins	
Vegetable	Simulated gastrointestinal digestion	↑	-	↑	-	[52]
	Simulated intestinal absorption	↓	-	↑		
Apple	Simulated gastric digestion	↓	↓	↓	The glycoside bond is hydrolyzed	[56]
	Simulated intestinal digestion	↓	↑	↑	High recovery of total dihydrochalcone (mainly phenyl propyl) at this stage (150.94% and 90.97%)	
	Simulated intestinal absorption	↓	↓	↓	The total phenol content was the lowest	
The Cinnamomum camphora seed kernel	Simulated gastrointestinal digestion	↓	↓	↓	Interactions with other compounds or changes in molecular structure affect its solubility	[69]
Wafers Enriched with Freeze-Dried Raspberry Pomace	Simulated gastrointestinal digestion	↑	↑	↑	Simulating the digestion process helps to release potentially bioavailable phenolic compounds from the food substrate	[70]

↑ indicates increase after treatment, ↓ indicates decrease after treatment.

Szymanowska observed that XOR inhibition was significantly higher in the simulated digestion treatment than in the PBS extraction group [70]. XOR inhibition was also increased after the simulated gastrointestinal digestion of cardamom and coffee, which were 3.04 and 1.02 times the EC50 (the extract concentration which provides 50% enzyme inhibitory activity) of the water extract, respectively [71]. In spices, the polyphenol contents of pepper, marjoram, basil, thyme, and ginger were found to increase after digestion in vitro, and the XOR inhibitory activity increased significantly in correlation to their increasing contents [66]. Likewise, the XOR inhibitory activity of Emblica extract was significantly improved after digestion in gastric and intestinal fluid [72]. Similar conclusions were also found in studies of vegetables (lettuce, tomato, onion, and garlic), coffee beans, and whole-wheat bread [67,71,73].

Collectively, the elevated XOR inhibitory activity of polyphenols during the gastrointestinal digestion phase can be attributed to the following reasons: (1) The chemical action of strong acids in digestion and the physical action of the mechanical movement destroys plant cell walls, resulting in the release of polyphenols from the food matrix; (2) The presence of pepsin inhibits the binding of polyphenols with proteoglycans and other macromolecules, and improves their bioavailability; (3) During digestion and absorption, the glycosidic bond of flavonoids is broken and the aglycone form, with its good inhibitory activity, is detached. The reason for decreases in inhibitory activity during intestinal digestion is that polyphenols are decomposed in large quantities, resulting in the loss of their active structures.

#### 3.2.2. In Vitro Absorption

The consequences of the decomposition and transformation of polyphenols during intestinal absorption are uncertain. The XOR inhibitory activity of spice polyphenols was found to increase after intestinal absorption, with a significant positive correlation with the content of flavonoids in their components (R = 0.81) [66]. In vitro, the absorption of wheat bread containing various amounts of onion polyphenols had a greater inhibitory effect on XOR than untreated components (BEF = 1.48–1.64, BEF means biological efficiency factor, inhibition of xanthine oxidase by buffered extracts/inhibition of xanthine oxidase by extracts after simulated intestinal absorption) [67]. In Gawlik-Dziki’s study, the inhibitory activity of garlic on XOR was significantly improved after gastrointestinal digestion and absorption, and the BEF of garlic enteric absorption components (=fm50 of simulated enteric absorption extract/fm50 of PBS extract) was as high as 5.56. Further, the XOR inhibitory activities of tomato and onion intestinal absorption components were also shown to increase (BEF = 1.85, 1.95); however, the XOR inhibitory activities of lettuce intestinal absorption components were significantly decreased (BEF = 0.65) [73]. Apple phenolics retained only 83% of their XOR inhibitory activity in the undigested fraction after intestinal absorption in vitro, reflecting their lowest total phenolic content. More studies are needed to ascertain the effects of polyphenol conversion at the intestinal absorption stage on biological activity [56].

Certain gaps in the scientific understanding of in vitro polyphenol digestion require in-depth exploration. There has been little research on the oral digestion stage, for example; however, it is known that albumin, mucin, and proline-rich proteins in saliva may impact the digestibility and absorption of particular polyphenols [74]. The bioaccessibility of polyphenols is impacted by tannins’ propensity to precipitate with proteins through hydrogen bonding and hydrophobic contact. In addition, the combination of protease and tannin in the mouth may also impact downstream gastrointestinal digestion. Moreover, during intestinal absorption, polyphenols are often absorbed in the colon, where some are decomposed by coliform bacteria; the biological activity mediated by intestinal flora is also a hot topic of research. Polyphenols can be used as prebiotics to maintain the balance of intestinal flora composition and promote the decomposition of purine and uric acid by intestinal flora, thus reducing hyperuricemia [75]. For example, the flavonoids extracted from saffron by-products show good uric acid-lowering effects while improving intestinal flora disorders in the hyperuricemia state [76]. Nagar pointed out that in the in vitro digestion study of polyphenols, an excess of dissolved oxygen and a lack of bile in the model may account for the low bioavailability of polyphenols [77].

### 3.3. Synergistic Effect of Polyphenols on XOR Inhibitory Activity

After polyphenols enter the systemic circulation, a variety of monomers typically act together with XOR. In recent years, scholars have discovered the synergistic effect between different polyphenol extracts to enhance the inhibitory effect of monomers on XOR. For example, in wheat bread supplemented with coffee bean extract, the extract showed a slight synergistic effect (CI = 0.92, CI means the combination index, the sum of the dose of drugs that provide inhibition in a combination), which was enhanced during in vitro digestion (CI = 0.43) [78]. A similarly synergistic effect has also been reported between coffee and cardamom extracts (IF = 0.71, IF means the interaction factor, which is the measured activity of a mixture of samples/theoretically calculated mixture activity) [71].

Interestingly, the structures of the other synergistic polyphenols screened from extracts are similar to the study of interaction from the perspective of structural analogs. The strongest synergistic effects on XOR inhibition were all shown to occur at low concentrations. The effect of synergistic effect on the ability of polyphenols to inhibit xanthine oxidase is shown in Table 3.

#### 3.3.1. Synergistic Inhibition of Polyphenol Monomers

In a study of the inhibitory mechanism of kaempferol on XOR, the best synergistic inhibitory effect with kaempferol and luteolin appeared at the concentration ratio of 1:10 (V_ab_ − V_c_ = −0.18), and it was speculated that the synergistic mechanism was caused by the conformational change of XOR during the competitive occupation of the binding site of xanthine. The best synergistic inhibition between kaempferol and morin (V_ab_ − V_c_ = −0.25) occurred at 1:1 (both concentrations were 2.0 × 10^−6^ mol L^−1^), and it was speculated that morin interacts with amino acid residues near the XOR hydrophobic cavity, while kaempferol binds to the active site and synergies. The synergistic effect of luteolin and kaempferol requires lower concentrations (0.5 to 1.0 × 10^−6^ mol L^−1^) [30]. Li conducted a screening and interaction study of the XOR-inhibitory polyphenols in Flos Sophorae Immaturus and found that only kaempferol and quercetin (1:1, 0.02 mg/mL) showed a subadditive effect. This was speculated to be caused by the difference between the competitive inhibitory effect of kaempferol and the mixed inhibitory effect of quercetin [79]. Kaempferide and galangin, which had the best XOR inhibitory activity, had the best synergistic inhibitory effect on XOR at the molar ratio of 1:4, and the mixture of kaempferide and galangin increased its affinity for XOR, showing competitive inhibition from both monomers [21]. In a study of chrysin and its structural analog apigenin, a synergistic effect was observed at a low concentration ratio of 2:1. Although both act as competitive inhibitors, the amino acid residues that bind at the active site are different and may be responsible for the synergistic effect [26].

In conclusion, two polyphenols with synergistic interactions usually have different binding sites to the enzyme and can bind to the enzyme simultaneously. Multiple polyphenols have more interaction sites with residues when bound to an enzyme than when a single polyphenol binds to an enzyme, and the enzyme’s conformation changes more when bound. By occupying the active site or changing the conformation of the enzyme, the substrate is prevented from binding to the enzyme [80]. In addition, the binding of certain polyphenols to enzymes promotes the binding of other monomers to enzymes. When one monomer binds to the enzyme, it alters the hydrophobicity of the enzyme, exposing the active site and making it easier for the remaining polyphenols to bind to the enzyme or promote more hydrogen bonding [81]. This is the reason why synergistic polyphenols have a better inhibitory effect on enzymes than the simple sum of the effects of the polyphenols acting alone. A certain degree of synergistic effect can, thus, promote the XOR inhibitory activity of monomer B by adding monomer A with an optimal inhibitory ratio.

#### 3.3.2. Polyphenols Synergistically Enhance Allopurinol

In addition to the interactions between polyphenol monomers, they can also synergistically enhance the inhibitory activity of allopurinol. Both baicalein and baicalin were shown to inhibit XOR in combination, and their molecular docking showed that they were bound to the FAD site, with a good synergistic inhibitory effect when combined. The combination of baicalein and allopurinol showed moderate synergistic effects at 50% and 70% inhibition levels, while baicalin and allopurinol showed a synergistic effect only at 50% inhibition levels (CI = 0.85) [24]. EGCG and GCG acted on XOR with combined inhibition, and EGCG and allopurinol showed synergistic effects at all levels of inhibition, although GCG and allopurinol showed a stronger synergistic effect of CI < 0.9 at all inhibition levels [25]. The synergistic effects of polyphenols and allopurinol may also achieve the same inhibitory effect, enabling the reduction of allopurinol dosage, which is beneficial to reducing the toxic effect of drugs on the body.

There is a need for a consistent measuring standard and evaluation system, and current studies on the interaction of polyphenols on XOR inhibition require additional research in terms of mechanism.

## 4. Conclusions

In this review, we summarized the structure–activity relationship of flavonoids previously shown to exhibit the best XOR inhibitory activity among polyphenols. Flavonoids are often bound to the MOC or FAD active sites of XOR, the key residues of which play roles in the polyphenol binding process through complex orientation, hydrogen bonding, π-π interactions, and the tightening of active pockets after binding. The hydroxylation of C5 and C7 positions in the A-ring of flavonoids contributes greatly to inhibitory activity. Hydroxyl groups at special sites can form hydrogen bonds with key residues of XOR, thereby improving the stability of flavonoid binding with enzymes. Moreover, methoxy substitution increases the polarity of the compound, improving its force of interaction with the hydrophobic residues in the enzyme, increasing inhibition ability. Glycosylation increases the steric hindrance of flavonoids, which is not conducive to entry into the active center of XOR, and significantly decreases their inhibitory ability. The planar structure is also an important prerequisite for the XOR inhibitory activity of flavonoids.

On the basis of this structure–activity relationship, changes in the XOR inhibitory activity of polyphenols in terms of several key factors, as well as their possible causes, were discussed. Appropriate heat treatment during processing and gastrointestinal digestion after ingestion may enhance XOR inhibitory activity by increasing polyphenol leaching, promoting flavonoid glycosidic bond breaking, and decreasing spatial site resistance. However, the changes that occur during intestinal absorption are uncertain and more research is required to clarify this stage. Polyphenols with similar structures can exert synergistic effects at low concentrations, and polyphenols can also synergistically enhance allopurinol, which may be conducive to reducing the toxic effects of drugs on the body and, thus, offers potential for future research and development.

## 5. Limitations and Future Outlook

A more in-depth understanding of the structure–activity relationship of polyphenols in the inhibition of XOR, as well as the influence of key factors on this activity, will aid in the discovery of natural products rich in polyphenols with high XOR inhibitory activity and provide support for the development of products that can alleviate hyperuricemia. However, there are some limitations to current studies:

1. The accuracy of various experimental methods differs and may lead to false positives in terms of XOR inhibitory activity. For example, the absorption peak of flavonoids at 280 nm interferes with the measurement of uric acid production at 295 nm, and measurement using UV–visible light produced significant deviations. A more accurate method, such as high-performance liquid chromatography (HPLC), could be used to calculate the inhibition ability of polyphenols on XOR, to further the analysis of their structure–activity relationship.
2. The structure–activity relationship of non-flavonoids in their inhibition of XOR requires further investigation. Our current summary of the structure–activity relationship has focused mainly on flavonoids; however, plant extracts are mostly polyphenol mixtures, so it is necessary to ascertain any differences between the structure–activity relationship of non-flavonoids (phenolic acids, chalcones, etc.) in their inhibition of XOR and that of flavonoids.
3. There are few studies on the changes in the activity of polyphenols during intestinal absorption, and the in vitro digestion and absorption models require optimization. Current in vitro digestion models lack consideration in terms of important influencing factors, such as salivary albumin, dissolved oxygen, and bile, which may lead to deviations in study conclusions. In addition, the simple dialysis simulation of the intestinal absorption process is very different from the real situation in vivo, which should be further explored using cell models.
4. Systems of assessment in interaction studies of different polyphenols differ greatly. It is difficult to evaluate synergistic effects in different evaluation models, and the exact mechanism of interaction requires additional investigation. Furthermore, interactions between polyphenols and other macromolecules in the food matrix (such as proteins, polysaccharides, and lipids) may impact the inhibitory efficacy of XOR through changes in their bioaccessibility.

## Figures and Tables

**Table 1:** Influence of processing factors on the xanthine oxidase inhibition ability of polyphenol extracts.

Treatment	Polyphenol Source	Optimal Condition	Total Polyphenols	Total Flavone	XOR Inhibition	Mechanism	Reference
Hydrothermal	Flos Sophorae Immaturus	Hydrothermal treatment at 220 °C for 30 min	↑	↑	↑	At high temperatures, polyphenols were dissolved and transformed	[49]
Stir-frying	Flos Sophorae Immaturus	At 240 °C for 60 min	-	-	↑	Polyphenols gradually increased after stir-frying treatment, which improved the xanthine oxidase inhibitory activity of FSI	[50]
Ultrasound-assisted heating treatments	Flos Sophorae Immaturus	Ultrasound (480 W, 60 min)-assisted heating treatments (180, 60 min)	↑	↑	↑	Ultrasonic treatment and heating treatment have synergistic effects, and the leaching and conversion of polyphenols can be promoted at high temperatures	[51]
Roasting	Caffeic	140 °C and 170 °C reactions, with 170 °C being optimal	-	-	↑	Oligomers with the strongest XOR inhibitory activity can be produced at 140–170 °C	[52]
Different solvent (n-Hexane, Chloroform, Methanol, Water)	Bryophyllum pinnatum	Methanol extract	-	-	88.30%	-	[53]
Different extraction conditions	Sunflower head	Ethanol (54.5%), temperature (72.3 °C), and time (138.8 min)	-	-	60.04%	The presence of different concentrations of bioactive compounds	[54]
Different techniques of extraction using four solvents	Artemisia campestris	Maceration with Ethyl acetate	322.1 ± 2.9 mg GAE/g DE	296.3 ± 0.3 mg GAE/g DE	5.0 ± 2.7 mg/L	-	[55]
High-pressure processing	Apple	-	↑	-	↓	The decrease in flavonol content after HP treatment may be related to the increase in soluble fiber in apples and the hindrance of quercetin release in fruit pulp	[56]

↑ indicates increase after optimal condition, ↓ indicates decrease after optimal condition.

**Table 3:** Synergistic effects of different polyphenols on the activity of XOR.

Combination		IC_50_	Inhibition Type	Binding Site	Proportion	Synergistic Concentration	Synergy Degree	Mechanism	Reference
Chrysin		1.26 ± 0.04 μM	Competitive	MOC	1:2	0.5 μM	V_ab_ − V_c_ = −0.12	Although it is combined in the same active cavity, it has a non-interfering position	[26]
Apigenin		3.57 ± 0.03 μM	Competitive	MOC		1.0 μM			
Kaempferol		2.18 ± 0.02 μM	Competitive	MOC	1:10	0.5 μM	V_ab_ − V_c_ = −0.18	Competitively binds to the active site	[30]
Luteolin		4.79 ± 0.02 μM	-	-		1.0 μM			
Kaempferol		2.18 ± 0.02 μM	Competitive	MOC	1:1	0.5 μM	V_ab_ − V_c_ = −0.25	Morin interacts with amino acid residues near the active chamber, while kaempferol directly binds to the active site	[30]
Morin		13.54 ± 0.02 μM	-	-		5.0 μM			
Kaempferide		48.25 μM	Competitive	-	1:4	2.0 μM	CI < 0.9	The binding of kaempferide and galangal alters the target amino acid residues and increases their binding affinity for XOR	[21]
Galangin		167.76 μM	Competitive	-		2.0 μM			
Quercetin		0.03 mg/mL	Mixed	-	1:1	-	V* − V_ab_ = 0.16	Kaempferol binds to the active center, while quercetin not only binds to the active center but also binds to the XOR–substrate complex	[79]
Kaempferol		0.11 mg/mL	Competitive	-		-			
Baicalein		7.54 ± 0.06 μM	Mixed	FAD	-	0.02 mg/mL	CI < 0.85	There are different binding postures, and the binding of baicalin can induce the conformational change of XOR, promote the binding of baicalein, and synergistically inhibit the activity of XOR	[24]
Baicalin		(1.23 ± 0.03) × 10^−4^ mol L^−1^	Mixed	FAD		0.02 mg/mL			
Chlorogenic		216.41 ± 32.48 μg/mL	Competitive	-	1:1	-	CI = 0.92	-	[78]
Ferulic		119.64 ± 25.45 μg/mL	Competitive	-		-			
Allopurinol	Fisetin	3.93 ± 0.12 μM	Mixed	FAD	-	70%	CI = 0.63	Different binding sites. In addition, the insertion of fisetin induces a conformational change in XOR that promotes allopurinol binding	[27]
	EGCG	40.50 ± 0.32 μM	Mixed	FAD	-	-	-	Different binding sites. Allopurinol binds to the active center of XOR, and EGCG and GCG bind to other active regions, making the binding between allopurinol and XOR more stable	[25]
	GCG	33.60 ± 0.53 μM	Mixed	FAD	-	-	-		
	Baicalein	7.54 ± 0.06 μM	Mixed	FAD	-	50%, 70%	CI < 0.9	-	[24]
	Baicalin	(1.23 ± 0.03) × 10^−4^ mol L^−1^	Mixed	FAD	-	50%	CI = 0.83		

MOC, molybdopterin cofactor; FAD, flavin adenine dinucleotide; V_ab_, true enzyme activity in the presence of two polyphenols; V_c_, the sum of the XO inhibition rates of two kinds of polyphenols, V_ab_ − V_c_ < −0.10, a synergistic effect is indicated; V*, the sum of the XO inhibition rates of two kinds of polyphenols; CI, the sum of the dose of drugs that provide inhibition in a combination, if CI < 1, a synergistic effect is indicated; EGCG, epigallocatechin gallate; GCG, gallocatechin gallate.

## Data Availability

The original contributions presented in the study are included in the article/Supplementary Materials, further inquiries can be directed to the corresponding author.

## Supplementary Materials

The following supporting information can be downloaded at: https://www.mdpi.com/article/10.3390/foods13152365/s1, Table S1: Chemical structure, xanthine oxidase inhibition, and interaction details of flavonoids.
