# Supplementary material for: Structure–Activity Relationships and Changes in the Inhibition of Xanthine Oxidase by Polyphenols: A Review
Source: Foods. 2024 Jul 26;13(15):2365. doi: 10.3390/foods13152365 (PMC11312107; doi:10.3390/foods13152365)
Supplement: Supplementary file 1 [file foods-13-02365-s001.zip › foods-3091876-supplementary.pdf]

Table S1. Chemical structure, xanthine oxidase inhibition, and interaction details of flavonoids.

Name	Substitutions		Inhibition*	IC50 (μM)	Experimental Method	Inhibition Type	Binding Site	Interaction Force With Enzyme Binding			Reference
	-OH	Others						Hydrogen Bond	Hydrophilic Force	π-π Force	
Kaempferide	3,5,7	4'-OCH_3	-	48.25	HPLC 290nm	competitive	MOC	Glu802、Thr1010、Ser876、Arg880	Leu648、Phe649、Met770、Leu873、Phe914、Phe1009、Val1011、Phe1013、	Phe914	[1]
Galangin	3,5,7	-	-	167.76	HPLC 290nm	competitive	MOC	Ser876、Asn768	Leu1014、Aal1078、Ala1079、Tyr1121	Phe914	
Luteoloside	5,4',5'	7-o-glucoside	-	-	UV-vis 295nm	N	MOC	Arg880、Lys771	Leu873、Phe914、Ala1079、Glu802、Val1011、Pro1076、Phe649、Phe1013、Leu648、Leu1014、Thr1010、Ser876、Phe1009	-	[2]
Diosmetin	5,7,3'	4'-OCH_3	0.29	5.08 ± 0.48	UV-vis 295nm	competitive	MOC	Val1011，Thr1010，Ala1079，Alg880、Asn768	Leu848、Asn768、Glu802、Leu873、Ala1078	Phe914、Phe1009	[3]
Baicalein	5,6,7	-	2.98	7.54 ± 0.06	UV-vis 293nm	mixed	FAD	Arg426, Arg394、Glu263	-	-	[4]

Baicalin	5,6	7-o-glucuronide	48.62	123 ± 3	UV-vis 293nm	mixed	FAD	Leu807、Val654、Ile835、Asn869、Arg804、His665、Thr653	Phe775、Leu807、Ile666、Val654、Ile835	-	
EGCG	5,7,3',4',5'	3-gallate (cis)	38.21	40.50 ± 0.32	UV-vis 294nm	mixed	FAD	Tyr58、Ser307、Ser344、Ala304、Val345	-	-	[5]
GCG	5,7,3',4',5'	3-gallate (trans)	31.70	33.60 ± 0.53	UV-vis 295nm	mixed	FAD	Asp59、Trp283、Lys57、Ser344、Thr311、Lys310	-	-	
Chrysin	5,7	-	0.43	1.26 ± 0.04	UV-vis 290nm	competitive	MOC	Thr1010、Glu879、Ser876	Leu648、Phe649、Glu802、Leu873、Phe1013	Val1011、Phe1013	[6]
Fisetin	3,7,4',5'	-	1.55	3.93 ± 0.12	UV-vis 293nm	mixed	FAD	Gln144、Gln423、Ala338	-	-	[7]

Quercetin	3,5,7,4',5'	-	-	6.45 ± 0.26	UV-vis 292nm	mixed	MOC	Glu802、Thr1010、Val1011	-	Phe914	[8]
Quercetin-3rhamnoside	5,7,3',4'	3-o-rhamnoside	-	12.09 ± 0.65	UV-vis 292nm	competitive	MOC	Met770、Glu802、Thr1010、Asn768	-	-	
Naringin	5,4'	7-o-neohesperidoside	-	230 ± 4.6	UV-vis 546nm	-	-	-	-	-	[9]
Naringenin	5,7,4'	-	-	4.4 ± 0.2	UV-vis 546nm	-	-	-	-	-	
Kaempferol	3,5,7,4‘	-	1.22	2.18±0.02	UV-vis 295nm	competitive	MOC	Asn768、Luz1	Phe1013, Pro1076, Glu 802, Leu1014, Leu648, Ser1075	Phe1009、Phe914	[10]
Wogonin	5,7	8-OCH_3	> 50	＞200	UV-vis 295nm	-	-	-	-	-	[11]
Luteolin	5,7,4’,5‘	-	0.22	0.85		-	-	-	-	-	
Isorhamnetin	3,5,7,4'	5'-OCH_3	1.06	4.03		mixed	MOC	Thr1010 (2.02 Å)	Leu648、Asn768、Glu802、Ser876、Arg880、Val1011、Phe1013、Leu1014、Pro1076、Ala1079	Phe914 (face-to-face), Phe1009 (face-to-edge)	
Myricetin	3,5,7,3',4',5'	-	17.83	67.93		-	-	-	-	-	
Hyperoside	5,7,3',4'	3-o-galactoside	> 50	＞200		-	-	-	-	-	

Rutin	5,7,3',4'	3-o-rhamnosylglucoside	> 50	＞200	-	-	-	-	-
Vitexin	5,7,4'	8-c-glucopyranosyl	> 50	＞200	-	-	-	-	-
Rhoifolin	5,4'	7-o-neohesperidoside	> 50	＞200	-	-	-	-	-
Genistein	5,7,4'	-	> 50	＞200	-	-	-	-	-
Glycitein	7,4'	6-OCH_3	> 50	＞200	-	-	-	-	-
Daidzein	7,4'	-	> 50	＞200	-	-	-	-	-
Formononetin	7	4'-OCH_3	> 50	＞200	-	-	-	-	-
Biochanin A	5,7	4'-OCH_3	> 50	＞200	-	-	-	-	-
Calycosin	7,3'	4'-OCH_3	> 50	＞200	-	-	-	-	-
Sophoricoside	5,7	4'-o-glucopyranoside	> 50	＞200	-	-	-	-	-
Genistin	5,4'	7-o-glucopyranoside	> 50	＞200	-	-	-	-	-
Puerarin	7,4'	8-o-glucopyranoside	> 50	＞200	-	-	-	-	-
Liquiritin	7	4'-o-glucopyranoside	> 50	＞200	-	-	-	-	-
Hesperitin	5,7,3'	4'-OCH_3	11.01	41.93	mixed	MOC	Ala1079 (2.19 Å), Thr1083 (1.92 Å), Val1259 (2.17 Å)	Arg912, Gln1040, Val1081, Ser1082, Glu1261	Phe798 (face-to-edge)

Taxifolin	3,5,7,3',4 ,	-	> 50	＞200	-	-	-	-	-
Farrerol	5,7,4'	-	> 50	＞200	-	-	-	-	-
Neohesperidin	5,5'	2'-OCH_3 7-o-neohesperidoside	> 50	＞200	-	-	-	-	-
Dihydromyricetin	3,5,7,3'.4'.5'	-	> 50	＞200	-	-	-	-	-
Epigallocatechin Gallate	5,7,3',4',5'	3-o-gallate	18.80	71.63	-	MOC	Gln767 (1.59 Å), Gln1040 (2.15 Å), Ala1079 (2.23 Å)	Arg912、Leu1042、Ala1078、Ser1080、Glu1261	Phe798 (face-to-face)

* MOC, molybdopterin cofactor; FAD, flavin adenine dinucleotide; HPLC, high performance liquid chromatography; UV-vis, ultraviolet–visible spectroscopy

1. Ou, R.; Lin, L.; Zhao, M.; Xie, Z. Action mechanisms and interaction of two key xanthine oxidase inhibitors in galangal: Combination of in vitro and in silico molecular docking studies. *International Journal of Biological Macromolecules* **2020**, *162*, 1526-1535, doi:10.1016/j.ijbiomac.2020.07.297.
2. Chen, J.; Wang, Y.; Pan, X.; Cheng, Y.; Liu, J.; Cao, X. Study on the interaction mechanism between luteoloside and xanthine oxidase by multi-spectroscopic and molecular docking methods. *Journal of Molecular Recognition* **2022**, *35*, doi:10.1002/jmr.2985.
3. Liu, Y.; Chen, H.; Xiang, H.; Lei, H.; Zhang, D.; Qiu, Y.; Xu, L. Inhibition and molecular mechanism of diosmetin against xanthine oxidase by multiple spectroscopies and molecular docking. *New Journal of Chemistry* **2020**, *44*, 6799-6809, doi:10.1039/d0nj00679c.
4. Zeng, N.; Zhang, G.; Hu, X.; Pan, J.; Zhou, Z.; Gong, D. Inhibition mechanism of baicalein and baicalin on xanthine oxidase and their synergistic effect with allopurinol. *Journal of Functional Foods* **2018**, *50*, 172-182, doi:10.1016/j.jff.2018.10.005.
5. Zhang, G.; Zhu, M.; Liao, Y.; Gong, D.; Hu, X. Action mechanisms of two key xanthine oxidase inhibitors in tea polyphenols and their combined effect with allopurinol. *J Sci Food Agric* **2022**, *102*, 7195-7208, doi:10.1002/jsfa.12085.
6. Lin, S.; Zhang, G.; Liao, Y.; Pan, J. Inhibition of chrysin on xanthine oxidase activity and its inhibition mechanism. *Int J Biol Macromol* **2015**, *81*, 274-282, doi:10.1016/j.ijbiomac.2015.08.017.
7. Zeng, N.; Zhang, G.; Hu, X.; Pan, J.; Gong, D. Mechanism of fisetin suppressing superoxide anion and xanthine oxidase activity. *Journal of Functional Foods* **2019**, *58*, 1-10, doi:10.1016/j.jff.2019.04.044.
8. Mehmood, A.; Li, J.; Rehman, A.U.; Kobun, R.; Llah, I.U.; Khan, I.; Althobaiti, F.; Albogami, S.; Usman, M.; Alharthi, F.; et al. Xanthine oxidase inhibitory study of eight structurally diverse phenolic compounds. *Frontiers in Nutrition* **2022**, *9*, doi:10.3389/fnut.2022.966557.
9. Cavia-Saiz, M.; Busto, M.D.; Pilar-Izquierdo, M.C.; Ortega, N.; Perez-Mateos, M.; Muñiz, P. Antioxidant properties, radical scavenging activity and biomolecule protection capacity of flavonoid naringenin and its glycoside naringin: a comparative study. *Journal of the Science of Food and Agriculture* **2010**, *90*, 1238-1244, doi:10.1002/jsfa.3959.
10. Wang, Y.J.; Zhang, G.W.; Pan, J.H.; Gong, D.M. Novel Insights into the Inhibitory Mechanism of Kaempferol on Xanthine Oxidase. *Journal of Agricultural and Food Chemistry* **2015**, *63*, 526-534, doi:10.1021/jf505584m.
11. Zhao, J.; Huang, L.; Sun, C.; Zhao, D.; Tang, H. Studies on the structure-activity relationship and interaction mechanism of flavonoids and xanthine oxidase through enzyme kinetics, spectroscopy methods and molecular simulations. *Food Chemistry* **2020**, *323*, doi:10.1016/j.foodchem.2020.126807.
